# Decision making and impulsiveness in abstinent alcohol-dependent people and healthy individuals: a neuropsychological examination

**DOI:** 10.1186/s13011-015-0020-7

**Published:** 2015-06-17

**Authors:** Natalie Körner, Peggy Schmidt, Michael Soyka

**Affiliations:** Algesiologikum GmbH, Heßstrasse 22, 80799 Munich, Germany; Privatklinik Meiringen, Postfach 612, Willigen, CH-3680 Meiringen Switzerland; Department of Psychiatry, Ludwig Maximilian University, Nussbaumstr. 7, 80336 Munich, Germany

**Keywords:** Decision making, Impulsiveness, Abstinence, Alcohol dependence, Iowa Gambling Task

## Abstract

**Background:**

Alcohol dependence is associated with deficits in decision making and increased impulsiveness. Therefore, we compared decision making in abstinent alcohol-dependent people (“abstainers”) and matched healthy individuals (“comparison group”) to determine whether impulsiveness or personality traits play a role in decision making.

**Methods:**

Abstainers (n = 40) were recruited from treatment facilities in and around Munich, Germany, and the comparison group (n = 40) through personal contacts and social media. We assessed decision making with the Iowa Gambling Task (IGT), impulsiveness with the Barratt Impulsiveness Scale (BIS-11) and personality traits with the NEO Five-Factor Inventory (NEO-FFI).

**Results:**

The comparison group performed significantly better in the IGT (mean profit € 159.50, SD 977.92) than the abstainers (mean loss - € 1,400.13, SD 1,362.10; *p* < .001) and showed significantly less impulsiveness in the BIS-11 (comparison group: mean 56.03, SD 7.80; abstainers: mean 63.55, SD 11.47; *p* < .001). None of the five personality traits assessed with the NEO-FFI differed significantly between the groups.

**Conclusion:**

The results confirm that abstinent alcohol-dependent people do not perform as well as healthy individuals in decision-making tasks and show greater impulsiveness, but in this study did not affect their decision-making ability.

## Introduction

Cognitive dysfunction and in particular deficits in executive functions such as inhibitory control processes may predispose an individual to alcohol misuse. Poor inhibitory control can be both a cause and consequence of excessive alcohol consumption (for review see [1]). Poor decision making may play an essential role in this respect [2]. Decision making is based on available or appropriated knowledge about situations, actions, courses of action and outcomes [3, 4]. The key to successful decision making is knowing the difference between options that provide advantageous results and those that provide disadvantageous results [5]. In this context Damasio postulated [3, 4] his “Somatic Marker Hypothesis” (SMH), which proposes that human judgments are influenced by *somatic markers* [3, 4, 6]. “Somatic” refers to a reaction relationship between body and brain and is a hallmark of affective and emotional responses [7]. Previous research has shown that the ventromedial prefrontal cortex (vmPFC) plays a crucial role in decision making, particularly in linking certain situations and emotional states and in evaluating them as good or bad for the individual [8, 4]; people with vmPFC lesions have been shown to have impaired decision making. The memory capability of the vmPFC is especially important if the memories relate to situations with unclear outcome and if the decision-making processes are dependent on previous experiences [4]. If these experiences or somatic markers are missing, options and results are rated as almost the same and the decision-making process is based purely on logical operations, which are considerably slower and more prone to error than personal experiences [4]. The ability to select the most advantageous option from a plurality of available options is particularly important for decision making [3]. Such a lack of foresight and complete failure to learn from mistakes is also known as myopia for the future [7, 9, 3]. From a political standpoint a better understanding of the psychological and neurobiological basis of alcoholism may help to develop better prevention and therapeutic strategies.

The Iowa Gambling Task (IGT) was developed by Bechara and colleagues [10] on the basis of research into the ability to create a balance between immediate rewards and long-term negative consequences [11]. Bechara and colleagues [12] supposed that an important factor for the development of substance abuse is a possible sub-function of the vmPFC that reduces access to somatic markers and the ability to remember that certain decisions are associated with negative consequences, such as future harmful consequences of repeated substance use [9]. Previous research into alcoholism showed that after long-term alcohol abuse the prefrontal cortex in particular is prone to lesions [13]. Accordingly, alcohol-dependent people performed worse than a comparison group in the IGT and showed less advantageous decision making [14–19], although this effect was not found for all alcohol-dependent people [20, 21]. A comparison of abstinent and relapsed alcohol-dependent people indicated that abstinent alcohol-dependent people (abstinent for up to 3 months) performed better in the IGT than non-abstainers [22] or briefly abstinent alcohol-dependent people [21]. These findings indicate that the duration of abstinence has a positive effect on decision making [21], although a study by Fine *et al.* [16] found that permanently abstinent alcohol-dependent people still performed worse in the decision task than the comparison group [15, 17, 21, 23]; of interest is that the abstinent alcohol-dependent people were able to maintain their abstinence despite their impaired decision making. Some studies found a learning effect in decision making in abstinent alcohol-dependent people – *i.e.,* the abstinent alcohol-dependent people learned by the last card in the IGT that there are disadvantageous decks [17, 21] – but other studies did not [15, 18].

Impulsivity is also of great importance in alcohol dependence because dependence is often associated with choosing smaller, immediate rewards rather than larger, delayed rewards [24], *i.e.,* alcohol dependence is in part a result of a series of impulsive decisions [25]. Thus, alcohol-dependent people show deficits in decision making and perform worse in the IGT than a comparison group [22]. Individuals with impulsive characteristics appear to skip the process of forming a preference from various options and to choose the first option that comes into their heads, without weighing up the pros and cons of the possible options [5]. Current and abstinent alcohol-dependent people showed higher impulsiveness values than a comparison group [25]. Abstinent individuals who relapsed after three months showed more impulsiveness than still abstinent alcohol-dependent people and performed worse in the IGT because they chose a higher percentage of disadvantageous cards [22]. Low impulsiveness has been found to be associated with better decision making and better results in the IGT [26]. Bechara [27] claims that decision making constitutes a dilemma that has to be solved by assessing various options, results and strategies, whereas impulse control has only one correct solution, namely to inhibit an overwhelming response [27]. Substance-dependent people show deficits in impulse control or decision making or both. For example, the choice between consuming the daily dose of alcohol and submitting to family pressure not to drink is a dilemma and the decision for the more favourable option depends on an intact decision-making ability, whereas the willpower not to buy new alcohol at the supermarket is based on response inhibition and the ability to control the impulse to buy alcohol [27].

Of further interest in this context are the five known personality traits, the Big Five: neuroticism, extraversion, openness to experience, agreeableness and conscientiousness [28]. In particular, research into alcoholism has shown that abstinent alcohol-dependent people who relapse within one year after detoxification have higher neuroticism and lower conscientiousness [29]. To date, barely any research has examined whether the Big Five affect decision making in abstinent alcohol-dependent people compared to healthy individuals.

This study aimed to compare decision-making skills in abstinent alcohol-dependent people (“abstainers”) and healthy individuals (“comparison group”). We hypothesized that the comparison group would score significantly higher on the IGT and would show a learning effect between the five blocks of the IGT. We also expected to find sex differences in IGT performance, because previous results for male and female adults showed a significant difference in performance: men chose on average more advantageous cards and thus achieved a higher profit in the long term than women [30]. This study also investigated whether impulsiveness differs between the abstainers and comparison group and whether it affects decision making. We hypothesised that impulsiveness (measured with the BIS-11) differs significantly between the abstainers and comparison group, that the scores in the three BIS-11 scales correlate with performance in the IGT and that total impulsiveness explains variance in performance in the IGT. As mentioned above, the presumed influence of personality traits on decision making has been little explored. Therefore, this study used the NEO Five-Factor Inventory (NEO-FFI) to examine the hypothesis that the abstainers show different personality traits than the comparison group and that the NEO-FFI personality traits explain the variance in IGT performance. Finally, we examined several additional factors such as intelligence, attention, depression, anger expression and hostility to see whether they differ between the abstainers and comparison group and whether they affect decision making, measured with the IGT.

## Methods

### Participants

In March to July 2013, a total of 87 individuals were enrolled in the study, 40 abstainers and 47 healthy individuals (comparison group). The abstainers (abstinent for ≥ 2 weeks) were recruited from the following institutions: ward C4 for addiction disorders at the Department of Psychiatry and Psychotherapy, Ludwig Maximilian University, Munich (LMU); outpatient clinic for addiction disorders (KPB) in Munich and Dachau; Blue Cross Munich; and Alcoholics Anonymous (AA), Munich. The abstainers had an ICD-10 diagnosis of dependency syndrome currently abstinent (F10.20) or dependency syndrome currently abstinent but in a sheltered environment (F10.21). These diagnoses are similar to the DSM-IV-TR diagnosis of alcohol dependence (303.90). The exclusion criteria included severe mental disorders such as psychosis, head injuries, organic brain syndrome, other cognitive disorders and a history of seizures (epilepsy).

The comparison group consisted of friends, colleagues and family members of the main author (NK) and people recruited *via* social media. Exclusion criteria for the comparison group were mental retardation, diagnosed learning disability, mental illness, substance addiction, illnesses of the central nervous system and neurological disorders.

The two groups were matched at the individual level in a 1:1 ratio (by hand) to make them comparable and reduce confounding (matched-pairs case–control study). Abstainers were matched to a participant in the comparison group on the basis of age, gender, highest school leaving certificate, years of education and marital status. As a consequence of matching, 7/47 healthy individuals in the comparison group were excluded because they could not be matched to an abstinent alcohol-dependent person.

The study was approved by the ethics committee of the University of Munich. Every participant provided written informed consent before the start of the study.

### Measurements and coding

Each participant performed the following study procedures once. The procedures were explained in detail and took a total of about 90 min for each participant to complete.

#### Iowa Gambling Task (IGT)

**Fig. 1:**
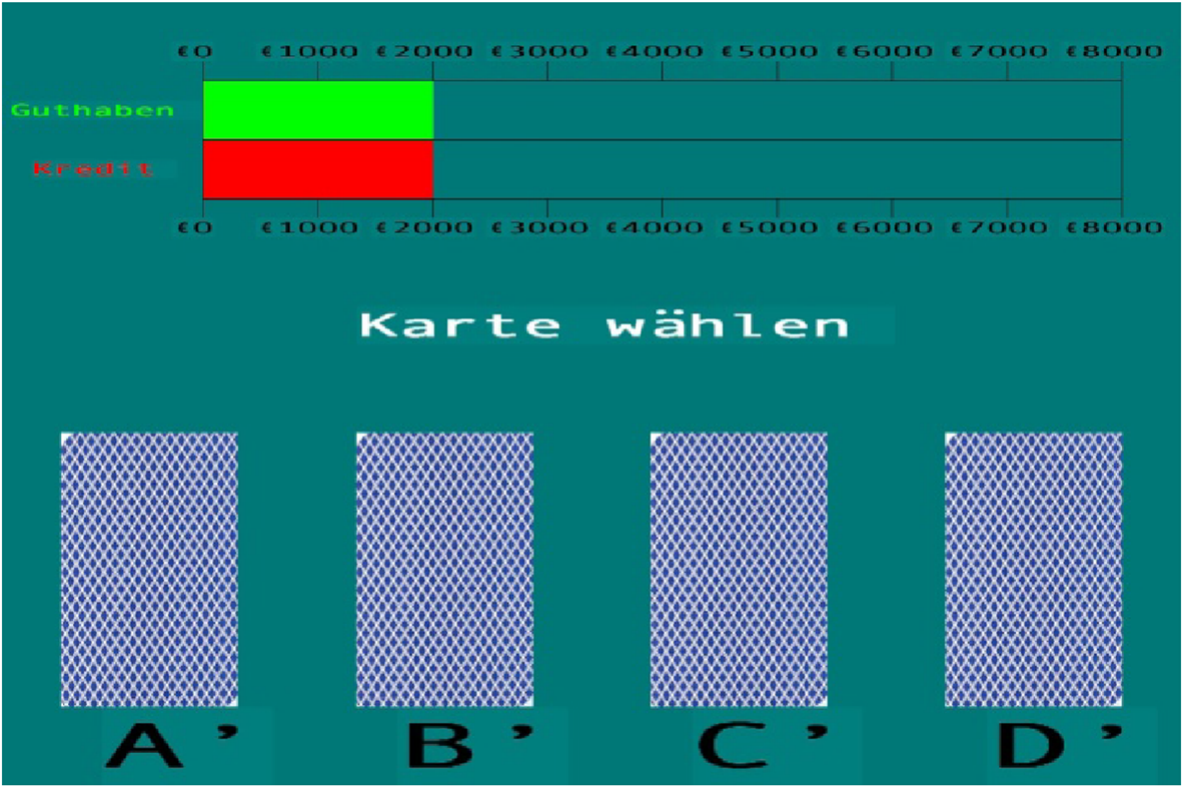
Computerized version 2.0 (German Version) of the Iowa Gambling Task (IGT). Guthaben: Profit. Kredit: Borrowed. Karte wählen: Choose a card. **a** deck A **b** deck B **c** deck C **d** deck D

The IGT was first published by Bechara and colleagues [10] as a neuropsychological task that realistically simulates decision making. The basic concept of IGT was developed to test the ability to create a balance between immediate rewards and long-term negative consequences and thus included punishment, reward and uncertainty about the consequences of a decision [10]. We used the computer-based German version of the IGT for adults aged 18–79 years [31]. In this task, participants are presented 4 decks of cards (A, B, C & D) on the screen and given a starting balance of € 2000 play money. At each turn (100 × totals) they select one card that is either a pure profit or a combined profit and loss (penalty) that can even exceed the amount of profit. The goal is to maximize the profit. Participants can see their current balance, represented by a green bar, and the amount they have lost, represented by a red bar (see Fig. 1). Participants do not know that playing from decks A and B results in an overall loss, despite higher rewards, and playing from decks C and D results in an overall gain. Each of the decks varies in relation to the immediate profit, the expected long-term earnings and the potential losses. For our study, we created five net scores from the 100 cards (20 cards each), which we referred to as blocks 1 to 5 [32]. The first block contained 20 cards, the first 10 cards of which included no loss. We calculated the net score for each block as (C + D) - (A + B) and then combined the five individual net scores to give a total score representing the overall IGT performance. All five scores could be either positive or negative. To obtain a net positive score, the participant had to choose more often from the advantageous decks C or D.

#### Barratt Impulsiveness Scale 11 (BIS-11)

The BIS-11 is a self-report instrument that assesses impulsive behaviour by rating over 30 items on a four-point scale (1–4). We used the German version of the scale by Preuß et al. [33] and measured the three dimensions of the second order (motor, non-planning and attention impulsiveness) and the total impulsiveness score.

#### NEO Five-Factor Inventory (NEO-FFI)

We used the second edition of the German version of the multidimensional personality questionnaire NEO-FFI by Borkenau and Ostendorf [28] to rate a total of 60 items in 5 dimensions (neuroticism, extraversion, openness to experience, agreeableness and conscientiousness) on a five-point scale (0–4).

#### Other assessments

In addition to the above ratings, we assessed attention with the d2 [24], intelligence with the multiple-choice word test MWT-B [27], depression with the Beck Depression Inventory II (BDI-II; [34]), state and trait anger with the State-Trait Anger Expression Inventory (STAXI; [35]) and hostility and aggression with the Buss-Durkee Hostility Inventory (BDHI; [36]). We used the EuropASI [37] to evaluate the severity of participants’ addiction and to record sociodemographic information. We used the validated German versions of all measurements.

### Statistical analysis

We analysed the reliability of all measurement instruments and performed several *t* tests for independent samples (group differences) in reference with Schafer and Kang [38]. Schafer and Kang [38] stated that matched groups should be regarded as independent (page 298) as they are not related. We used a between-subjects design in contrast to the within-subjects design of dependent samples.

We checked the variance homogeneity with the in the t-test procedure included levene-test and its test parameter “F”. To assume that the variances are homogenous, the F-value may not be significant. This condition was fulfilled. The distribution of mean differences was assumed to be approximately normally distributed at a total sample size > 50 (see [39]). We also performed correlation analyses, one-way and two-factorial analyses of variance and a two-factor ANOVA with repeated measures (learning effect) as well as multiple regressions with the inclusion method or forward method. To analyse whether the duration of abstinence might have affected our results, especially decision making, we divided the abstainers into a group with short-term abstinence (group A, abstinent for two weeks to six months) and one with long-term abstinence (group B, abstinent for more than six months) and compared the two.

## Results

The abstainers had been abstinent from two weeks to 38 years (mean 38.45 months, SD 87.37) and had a mean age of 48.15 years (SD 10.51, range 20–72 years). The mean age of the comparison group was 45.40 years (SD 10.73, range 23–77 years). Both samples included 13 women and 27 men; in each group, 12 participants were married, 7 divorced, 19 single, 1 separated and 1 widowed. The mean years of education among the abstainers was 15.15 years (SD 2.76, range 10–23 years) and among the comparison group 15.00 years (SD 2.70, range 9–22 years). In each group, 16 individuals had attended school to the end of year 9 or 10 (“Hauptschulabschluss” or “Realschulabschluss”) and 24 had attended school to the end of year 12 (“Fachabitur” or “Abitur”).

### Reliability analysis

Cronbach’s alpha varied between α = .57 and α = .91 for all measurements (see Table 1).

**Table 1:** Reliability analysis of all scales used in the questionnaires and tests in abstinent alcohol-dependent people (n = 40) and healthy individuals (n = 40)

	Number	Item number	Alpha
d2 – attention questionnaire	80	4	.69
BDI-II - depression	80	21	.86
IGT- performance (5 blocks)	80	5	.62
NEO-FFI – neuroticism	80	12	.90
NEO-FFI – extraversion	80	12	.85
NEO-FFI – openness to experience	80	12	.82
NEO-FFI – agreebleness	80	12	.65
NEO-FFI – conscientiousness	80	12	.89
BIS-11 – attention impulsiveness	80	8	.59
BIS-11 – motor impulsiveness	80	11	.69
BIS-11 – non-planning impulsiveness	80	11	.76
BIS-11 – total impulsiveness	80	30	.84
STAXI – anger-state	80	10	.91
STAXI – anger-trait	80	10	.83
BDHI – assault	80	10	.68
BDHI – indirect hostlity	80	9	.72
BDHI – irritability	80	11	.80
BDHI – negativism	80	5	.63
BDHI – recentment	80	8	.71
BDHI – suspicion	80	10	.73
BDHI – verbal hostility	80	13	.57
BDHI – guilt	80	9	.60

### Decision making

**Fig. 2:**
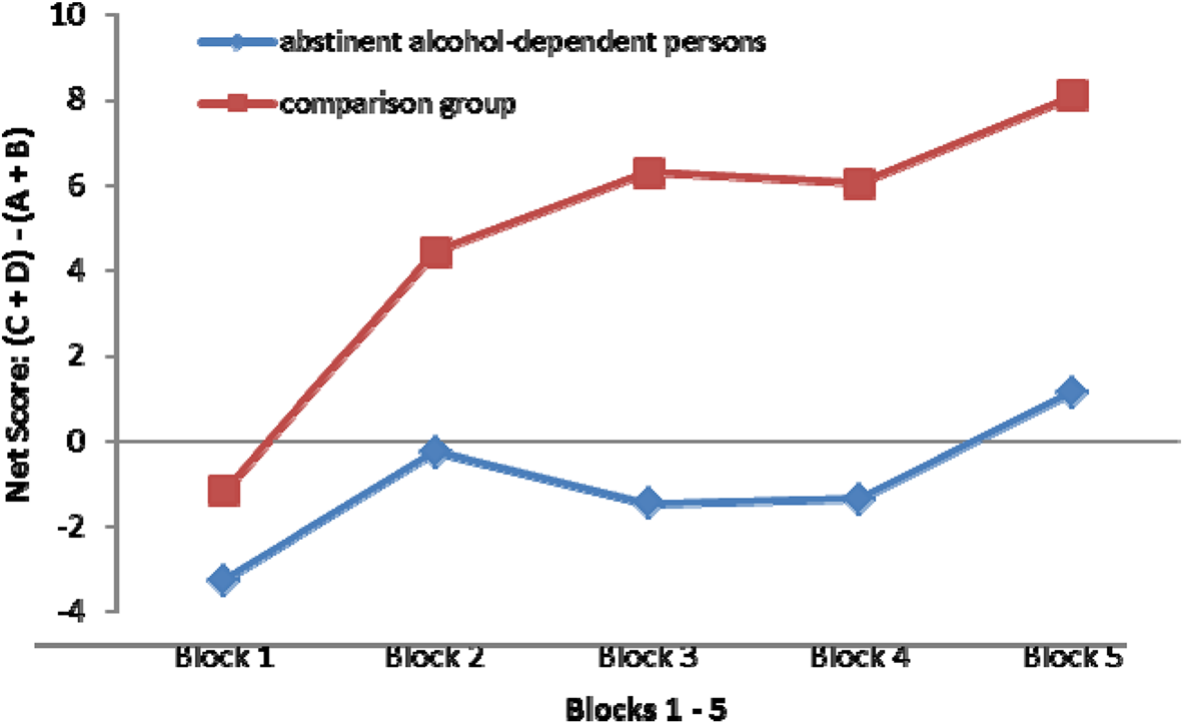
Mean net scores (see methods) of abstinent alcohol-dependent people (“abstainers”) and healthy individuals (“comparison group”) in the 5 blocks of the Iowa Gambling Task (IGT)

The comparison group earned a mean total profit (without the starting balance) of € 159.50 (SD 977.92) and the abstainers lost a mean of € -1,400.13 (SD 1,362.10), t(71) = 5.88, *p* < .001. Figure 2 shows the distribution of the means of the five blocks (20 cards each).

To evaluate whether a learning effect occurred across the five blocks of the IGT, we calculated a two-factor analysis of variance with repeated measures (2[Group affiliation] × 5[Blocks]). The between-subjects effects for group affiliation were significant (*F*(1,78) = 33.22, *p* < .001) and there was a significant main effect across all five blocks (*F*(3,218) = 9.38, *p* < .001), *i.e.,* IGT performance differed significantly between the abstainers and comparison group in each of the five blocks. The interaction effect for blocks x group affiliation was not significant (*p* = .10), *i.e.,* both samples had a similar profile over the 100 cards. There was a significant mean difference, especially for blocks 1 and 5 (mean difference [MD] -6.83, *p* < .001). The negative value reflects the fact that block 1 held lower values than block 5, confirming the learning effect from block 1 to block 5. Finally we used a two-factorial analysis of variance to test whether IGT performance differed between men and women in each group. The entire model explained 30.3 % (*F*(3,76) = 12.45, *p* < .001) of the total variance. While the main effect was highly significant for group affiliation (*F*(1,76) = 36.87, *p* < .001), the main effect for sex did not explain more variance and was not significant (p = .55). The comparison of the three independent samples short-term abstainers (group A), long-term abstainers (group B) and comparison group did not reveal a correlation between the duration of abstinence and decision-making abilities (see Table 2).

**Table 2:** Correlation between duration of abstinence and decision making in short-term abstainers (group A, alcohol-dependent people abstinent for 2 weeks to 6 months; n = 20) and long-term abstainers (group B, abstinent for more than 6 months; n = 20); p > .05 (2-tailed)

	Months		Decision making	
	A	B	A	B
Duration of abstinence (months)	1	1	-.35	.07
IGT performance (decision making)			1	1

### Impulsiveness

The total BIS-11 score was significantly higher in the abstainers (mean 63.55, SD 11.47) than in the comparison group (mean 56.03, SD 7.80) (*t*(69) = 3.43, *p* < .001). Table 3 shows the mean values of the three subscales of the BIS-11 and the level of significance of the group differences.

**Table 3:** Barratt Impulsive Scale (BIS-11) mean values, standard deviations and *t* values for all impulsiveness subscales in abstinent alcohol-dependent people (“abstainers”) and healthy individuals (“comparison group”)

	Abstainers (n = 40)	Comparison group (n = 40)	
	M (SD)	M (SD)	t value
Attention Impulsiveness	15.40(3.11)	13.83 (2.53)	2.48*
Motor impulsiveness	23.08 (4.69)	20.40 (3.55)	2.87**
Non-planning impulsiveness	25.08 (6.27)	21.80 (3.83)	2.82**
Total impulsiveness	63.55 (11.47)	56.03 (7.80)	3.43***

*M* mean, *SD* standard deviation

* *p* < .05; ** *p* < .01; *** *p* < .001.

To examine whether total impulsiveness explained additional variance in IGT performance, we performed a logistic regression analyses with a split sample (comparison group/abstainers) and the factors total impulsiveness as well as IGT performance. The total impulsiveness could not differentiate between abstainers and the comparison group according to their decision making (OR = 1.01; Wald = 3.43; 95%CI = 0.99-1.13; p = 0.06).

### Personality traits

We were unable to confirm our hypothesis that the abstainers and comparison group differed in their personality traits: none of the five NEO-FFI scales showed a significant group difference between the two samples, as determined with a *t* test for independent samples and a comparison of the two samples with the German norm values (see Table 4) [28].

**Table 4:** NEO Five-factor inventory mean values, standard deviations and a one-way analysis of variance with a split sample in abstinent alcohol-dependent people (“abstainers”) and healthy individuals (“comparison group”)

	Abstainers (n = 40)				Comparison group (n = 40)				German norm (*N* = 11.724)
	M (SD)	*β*	SE	corr. R^2^	M (SD)	*β*	SE	corr. R^2^	M (SD)
Neuroticism	20.03 (9.72)	-.02	0.46		16.03 (8.77)	.28	0.57		21.95 (8.36)
Extraversion	27.10 (7.98)	.13	0.61		28.90 (7.29)	.24	0.75		28.38 (6.70)
Openness to experience	30.43 (7.69)	-.09	0.42		29.70 (7.96)	.02	0.60		32.10 (6.48)
Agreebleness	30.10 (5.63)	.12	0.59		31.70 (4.73)	-.15	1.07		30.23 (5.69)
Conscientiousness	33.00 (8.24)	-.13	0.48		36.03 (6.84)	-.15	0.82		30.87 (7.13)
				-.108				-.015	

*M* mean, *SD* standard deviation, *β* (beta), *SE* (standard error), *corr. R* ^2^ (corrected R^2^)

*p* > .05

We determined the values for the five personality traits (neuroticism, extraversion, openness to experience, agreeableness and conscientiousness) in a one-way analysis of variance with a split sample (comparison group/abstainers) and found that none of them could explain a significant proportion of the total variance in IGT performance in either group (abstainers: *p* = .94; comparison group: *p* = .50).

### Other relevant factors

We used *t* tests for independent samples to evaluate whether abstainers differed significantly from the comparison group with respect to the factors assessed with the MWT-B (premorbid intelligence), d2 (attention), BDI-II (depression), STAXI (state and trait anger) and BDHI (hostility and aggression). Results are given in Table 5. The MWT-B showed no significant group difference (*p* = .10), so that intellectual abilities can be assumed to be comparable in both samples. The attention variables of d2 showed significant group differences for processing speed *t*(61) = 3.58, *p* < .01 and the quality of performance *t*(69) = 2.42, *p* < .05, but not for rule compliance (*p* = .07). The severity of depression showed no significant difference (*p* = .09). Furthermore, state anger (*p* = .21) and trait anger (*p* = .70), measured with the STAXI, did not differ significantly. The BDHI showed only two significant group differences, in suspicion *t*(78) = 2.21, *p* < .05, and guilt *t*(78) = 2.36, *p* < .05. None of the other BDHI factors differed significantly between the groups.

**Table 5:** Means, standard deviations and t values in abstinent alcohol-dependent people (“abstainers”) and healthy individuals (“comparison group”) of other factors such as intelligence, attention, depression, anger expression and hostility that may affect decision making, measured with the Iowa Gambling Task

	Abstainers (n = 40)	Comparison group (n = 40)	
	M (SD)	M (SD)	*t* value
MWT-B			
IQ – premorbid	114.60(14.82)	109.45(12.58)	1.68
d2			
Processing speed	62.65(33.77)	84.50(18.66)	3.58**
Rule compliance	62.40(26.72)	51.48(26.27)	−1.84
Quality of performance	67.38(29.88)	81.28(20.55)	2.43*
BDI-II			
Depression	8.63(8.32)	5.93(5.40)	1.72
STAXI			
State anger	1.25(0.41)	1.14(0.37)	1.27
Trait anger	1.90(0.51)	1.86(0.48)	0.38
BDHI – Factors			
Assault	0.34 (0.21)	0.29 (0.24)	1.01
Indirect hostility	0.42 (0.27)	0.48 (0.22)	−1.20
Irritability	0.43 (0.27)	0.45 (0.29)	−0.30
Negativism	0.51 (0.29)	0.45 (0.32)	0.89
Resentment	0.26 (0.25)	0.18 (0.20)	1.60
Suspicion	0.38 (0.25)	0.26 (0.23)	2.21*
Verbal hostility	0.46 (0.17)	0.44 (0.20)	0.51
Guilt	0.43 (0.21)	0.33 (0.20)	2.36*

*M* mean; *SD* standard deviation

*MWT*-*B* multiple-choice word test, *BDI*-*II* Beck Depression Inventory II, *STAXI* State-Trait Anger Expression Inventory, *BDHI* Buss-Durkee Hostility Inventory

*p* > .05; * *p* <. 05; ** *p* < .01

To evaluate whether one of the other relevant factors (MWTB, d2, BDI-II, STAXI and BDHI) may explain additional variance in IGT performance, we used a multiple regression with a forward procedure. We thereby used only the independent variables with the smallest probability of F (*p* < .05). Consequently, only one variable was included in the model for the abstainers, namely *negativism*. This variable explained 20.3 % (F(1,38) = 10.96, *p* < .01) of the IGT performance and thus explained decision making in the abstainers. None of the variables was eligible for inclusion in the model for the comparison group, *i.e.,* none of the calculated variables explained variance in IGT performance.

## Discussion

Impulsivity and impaired decision making are interesting neurocognitive conceptualizations that may in part explain the development and course of alcoholism. This study investigated the hypothesis that abstinent alcohol-dependent people (“abstainers”) on average have a higher level of impulsivity and perform worse in psychometric tests measuring decision-making tasks (IGT) than a matched comparison group of healthy individuals. The study found that the total profit, profit in the individual five blocks and the total IGT score were lower in the abstainers than in the comparison group. Thus, we were able to confirm our hypothesis that abstinent alcohol-dependent people show deficits in decision making. We were also able to confirm that the abstainers show higher impulsiveness scores than healthy individuals in the BIS-11. Personality traits did not differ between the abstainers and comparison group and did not explain variance in the IGT. Of the additional factors we examined, only the “suspicion” factor of the BDHI affected decision making in the abstainers.

The abstainers often chose the disadvantageous decks of the IGT, indicating that they show impairments in predicting the long-term negative consequences of their actions. While the comparison group were able to achieve gains in the IGT, the abstainers more often selected cards from the disadvantageous decks. Despite the visible green and red bars during the game, the abstainers made only very small modifications to their strategy. Group affiliation (abstainers *vs.* comparison group) explained 28.7 % of the total variance in IGT performance. These results are comparable with those of many studies in this research field (*e.g.,* [14, 15, 17, 22, 23]). Results for block 1 (20 cards, the first 10 of which did not include a loss) did not differ significantly between the two groups, indicating that initially the comparison group and abstainers behaved in the same way: both groups chose the card decks A and B more frequently [15] and the comparison group also preferred the higher profits and rewards (if no losses occurred). As the first losses began, comparison group changed their strategy, *i.e.,* in block 2 they preferred the advantageous cards and decided to lower their risk. The abstainers, on the other hand, changed their strategy later or not at all and did not distance themselves from risky decision making until block 5. This finding is in line with other studies (*e.g.,* [23]). There could be several explanations for the abstainers’ behaviour. First, the late change to choosing advantageous cards could be due to a lack of ability to remember by using somatic markers. In this case, the decisions would be purely based on logical but slower operations [4] and knowledge about the logic of the game would be delayed. Second, the abstainers might find it difficult to recognize the long-term negative consequences of disadvantageous cards for the final result of the game. Third, they might have lacked motivation to maximize profits because the earned profit was not paid and thus no immediate reward was recognizable. To counteract this third possibility, future studies could convert the € 2,000 starting balance of play money into € 2 real money. If the € 2 starting balance is lost and the game ends in the negative range, the participant gets nothing (indirect punishment). Otherwise, if the game ends in the positive range, for example at € 3,478 play money, the equivalent of € 3.48 is paid out to the participant at end of study (immediate reward). This option depends on the financial resources of the study. We did not give a monetary reward to participants in our study.

The abstainers did show an improvement from block 2 to block 5, *i.e.,* they showed a learning effect over the 100 cards [17, 21]. Despite this positive finding, the abstainers were slower than the comparison group to learn and change their strategy across the 5 blocks. In healthy individuals the learning effect is based on the memory capability of the vmPFC, which retrieves memories from past experiences (changing the deck of cards in case of loss) and applies them to the current situation [4]. The vmPFC is susceptible to damage after prolonged alcohol abuse, which would explain the worse performance and slower learning effect in our group of abstainers. Our finding confirms the hypothesis of Damasio [3] that lesions in this area of the brain lead to worse results in the IGT.

Within each sample, the men and women behaved similarly. The lack of difference between men and women could possibly be due to the unequal distribution of the sexes across the two groups, but it is in agreement with findings of other studies, *e.g.,* [12].

Abstainers showed higher impulsiveness scores than the comparison group in the three subscales of the BIS-11 and in the total score, which is in line with findings of other studies [22, 25]. These findings indicate that the abstainers more often acted impulsively (motorized), more often focused on the present than on the future (non-planning) and were more likely to have difficulty concentrating (attention). But this behaviour did not affect their IGT performance. These results are in line with those of Overman *et al.* [30], who could not demonstrate in a sample of young adults that making good or bad decisions concerning long-term outcome was dependent on the expression of impulsiveness. The higher impulsiveness of the abstainers in our study is not necessarily surprising. Previous research has already found that impulsive characteristics can be considered as risk factors for later dependence [40]. In our study, impulsiveness did not affect decision making in the comparison group. Higher impulsiveness has been shown to be associated with disadvantageous decision making in abstainers compared to a comparison group [26]. Studies have not yet investigated the effect of high and low impulsiveness on decision making.

We could not confirm our hypotheses that personality traits differ between abstainers and healthy individuals and that the five personality traits of the NEO-FFI explain variance in IGT performance (see also [41]). One could speculate that the higher extraversion of the abstainers results in riskier behaviour on the IGT or that the marked conscientiousness of the comparison group is related to their better decision making. Other studies [23, 41] used personality tests such as the Temperament and Character Inventory (TCI) to measure harm avoidance and novelty seeking, for example. Mueller *et al.* [41] found that abstinent patients have more persistence and show less novelty seeking than relapsed patients; the patients were not compared with comparison group. The lack of a correlation between personality and decision making in our study may have been due to the choice of the measuring instrument. For example, the TCI was shown to produce a more differentiated outcome in patients with alcohol dependence [41].

With respect to the BDHI factors, only “suspicion” had a significant effect on decision making in the abstainers. “Suspicion” explained 20.3 % of the variance in decision making in the abstainers, indicating that the ability to behave in an appropriate manner towards other people or authorities affects decision-making behaviour. This ability could possibly affect the decision for or against therapy or hinder the acceptance of help from other people, such as family members or authorities (*e.g.,* doctor or therapist). However, this is only a conjecture. The other tested factors may show only group differences, but they did not affect the decision making of the two samples.

Our study has some limitations. First, the sample size of 80 participants is rather small in comparison to previous studies in abstainers [16, 42]. Second, the lack of gender difference could be explained by the imbalance between women and men (13:27) in the total sample. Third, we accepted the diagnoses of abstinence made by the treatment institutions and did not re-evaluate abstinence with standardized diagnostic instruments. We do not doubt that the treatment facilities diagnosed abstinence correctly but would like to draw attention to the possibility that an additional review of abstinence (with gamma-glutamyltransferase or a breath alcohol test) would have increased the reliability of our study results.

When implementing the IGT, particular attention should be paid to the depth of instruction given. Balodis et al. [43] found a significant difference in IGT performance if sparse or detailed instructions were given. Detailed instructions, such as those of Bechara *et al.* [32], ensure better interpretability of the results. This topic may be of interest for future research.

Our findings support the hypothesis that healthy individuals are better at making decisions than abstinent alcohol-dependent people and that impulsiveness and other aspects such as quality of performance, processing speed and defiant behaviour towards other people (suspicion) are associated with impaired decision making. Additional studies should be performed to confirm our findings and further investigate the hypotheses that could not be confirmed, also in high-risk individuals and active drinkers. Early detection of individuals with deficits in decision making may help to introduce adequate preventive strategies to keep these individuals from starting to drink too much alcohol.

## References

[1] Lopez-Caneda E, Rodriguez Holguin S, Cadaveira F, Corral M, Doallo S (2014). Impact of alcohol use on inhibitory control (and vice versa) during adolescence and young adulthood: a review. Alcohol Alcohol..

[2] Norman AL, Pulido C, Squeglia LM, Spadoni AD, Paulus MP, Tapert SF (2011). Neural activation during inhibition predicts initiation of substance use in adolescence. Drug Alcohol Depend..

[3] Damasio AR (1994). Descartes’ error. Emotion, reason, and the human brain.

[4] Damasio AR (1996). The somatic marker hypothesis and the possible functions of the prefrontal cortex. Philos Trans R Soc Lond B Biol Sci..

[5] Byrnes JP (2002). The development of decision-making. J Adolesc Health..

[6] Damasio AR, Tranel D, Damasio H, Levin HS, Eisenberg HM, Benton AL (1991). Somatic markers and the guidance of behavior: theory and preliminary testing. Frontal lobe function and dysfunction.

[7] Bechara A (2005). Decision making, impulse control and loss of willpower to resist drugs: a neurocognitive perspective. Nat Neurosci..

[8] Bechara A (2004). The role of emotion in decision-making: evidence from neurological patients with orbitofrontal damage. Brain Cogn..

[9] Bechara A, Damasio H (2002). Decision-making and addiction (part I): impaired activation of somatic states in substance dependent individuals when pondering decisions with negative future consequences. Neuropsychologia..

[10] Bechara A, Damasio AR, Damasio H, Anderson SW (1994). Insensitivity to future consequences following damage to human prefrontal cortex. Cognition..

[11] Grant S, Contoreggi C, London ED (2000). Drug abusers show impaired performance in a laboratory test of decision making. Neuropsychologia..

[12] Bechara A, Dolan S, Denburg N, Hindes A, Anderson SW, Nathan PE (2001). Decision-making deficits, linked to a dysfunctional ventromedial prefrontal cortex, revealed in alcohol and stimulant abusers. Neuropsychologia..

[13] Dao-Castellana MH, Samson Y, Legault F, Martinot JL, Aubin HJ, Crouzel C (1998). Frontal dysfunction in neurologically normal chronic alcoholic subjects: metabolic and neuropsychological findings. Psychol Med..

[14] De Wilde B, Verdejo-Garcia A, Sabbe B, Hulstijn W, Dom G (2013). Affective decision-making is predictive of three-month relapse in polysubstance-dependent alcoholics. Eur Addict Res..

[15] Dom G, De Wilde B, Hulstijn W, van den Brink W, Sabbe B (2006). Decision-making deficits in alcohol-dependent patients with and without comorbid personality disorder. Alcohol Clin Exp Res..

[16] Fein G, Klein L, Finn P (2004). Impairment on a simulated gambling task in long-term abstinent alcoholics. Alcohol Clin Exp Res..

[17] Goudriaan AE, Oosterlaan J, de Beurs E, van den Brink W (2005). Decision making in pathological gambling: a comparison between pathological gamblers, alcohol dependents, persons with Tourette syndrome, and normal controls. Brain Res Cogn Brain Res..

[18] Kim YT, Sohn H, Jeong J (2011). Delayed transition from ambiguous to risky decision making in alcohol dependence during Iowa Gambling Task. Psychiatry Res..

[19] Mazas CA, Finn PR, Steinmetz JE (2000). Decision-making biases, antisocial personality, and early-onset alcoholism. Alcohol Clin Exp Res..

[20] Hildebrandt H, Brokate B, Hoffmann E, Kroger B, Eling P (2006). Conditional responding is impaired in chronic alcoholics. J Clin Exp Neuropsychol..

[21] Loeber S, Duka T, Welzel H, Nakovics H, Heinz A, Flor H (2009). Impairment of cognitive abilities and decision making after chronic use of alcohol: the impact of multiple detoxifications. Alcohol Alcohol..

[22] Bowden-Jones H, McPhillips M, Rogers R, Hutton S, Joyce E (2005). Risk-taking on tests sensitive to ventromedial prefrontal cortex dysfunction predicts early relapse in alcohol dependency: a pilot study. J Neuropsychiatry Clin Neurosci..

[23] Tomassini A, Struglia F, Spaziani D, Pacifico R, Stratta P, Rossi A (2012). Decision making, impulsivity, and personality traits in alcohol-dependent subjects. Am J Addict..

[24] Brickenkamp R (2002). Test d2: Aufmerksamkeits-Belastungs-Test (9., überarbeitete und neu normierte Aufl.).

[25] Petry NM (2001). Delay discounting of money and alcohol in actively using alcoholics, currently abstinent alcoholics, and controls. Psychopharmacology (Berl)..

[26] Upton DJ, Bishara AJ, Ahn WY, Stout JC (2011). Propensity for risk taking and trait impulsivity in the Iowa Gambling Task. Pers Individ Dif..

[27] Lehrl S (2005). Mehrfachwahl-Wortschatz-Intelligenztest MWT-B (5., unveränderte Auflage).

[28] Borkenau P, Ostendorf F (2008). NEO-Fünf-Faktoren Inventar nach Costa und McCrae (NEO-FFI). Manual (2., neu normierte und vollständig überarbeitete Auflage).

[29] Bottlender M, Soyka M (2005). Impact of different personality dimensions (NEO Five-Factor Inventory) on the outcome of alcohol-dependent patients 6 and 12 months after treatment. Psychiatry Res..

[30] Overman WH, Frassrand K, Ansel S, Trawalter S, Bies B, Redmond A (2004). Performance on the IOWA card task by adolescents and adults. Neuropsychologia..

[31] Bechara A (2007). Iowa gambling task professional manual.

[32] Bechara A, Damasio H, Damasio AR, Lee GP (1999). Different contributions of the human amygdala and ventromedial prefrontal cortex to decision-making. J Neurosci..

[33] Preuss UW, Rujescu D, Giegling I, Watzke S, Koller G, Zetzsche T (2008). [Psychometric evaluation of the German version of the Barratt Impulsiveness Scale]. Nervenarzt.

[34] Hautzinger M, Keller F, Kühner C (2006). BDI-II; beck depressions-inventar revision.

[35] Schwenkmezger P, Hodapp V, Spielberger CD (1992). Das State-Trait-Ärgerausdrucks-Inventar STAXI. Handbuch.

[36] Kornadt H, Buss AH, Durkee A (1982). Buss-Durkee hostility-guilt inventory – German version. Aggressionsmotiv und Aggressionshemmung. 1. Empirische und theoretische Untersuchungen zu einer Motivationstheorie der Aggression und zur Konstruktvalidierung eines Aggressions-TAT und andere aggressionsrelevante Verfahren.

[37] Gsellhofer B, Küfner H, Vogt M, Weiler D (1999). European addiction severity index: EuropASI; nach der 5. Aufl. der amerikanischen version von McLellan (1992) und der europäischen version des ASI; manual für training und durchführung.

[38] Schafer JL, Kang J (2008). Average causal effects from nonrandomized studies: A practical guide and simulated example. Psychol Methods.

[39] Bortz J (1989). Statistik für Sozialwissenschaftler.

[40] Joos L, Schmaal L, Goudriaan AE, Fransen E, Van den Brink W, Sabbe BG (2013). Age of onset and neuropsychological functioning in alcohol dependent inpatients. Alcohol Clin Exp Res..

[41] Muller SE, Weijers HG, Boning J, Wiesbeck GA (2008). Personality traits predict treatment outcome in alcohol-dependent patients. Neuropsychobiology..

[42] Cantrell H, Finn PR, Rickert ME, Lucas J (2008). Decision making in alcohol dependence: insensitivity to future consequences and comorbid disinhibitory psychopathology. Alcohol Clin Exp Res..

[43] Balodis IM, MacDonald TK, Olmstead MC (2006). Instructional cues modify performance on the Iowa Gambling Task. Brain Cogn..

